# Human-centered design in the context of social determinants of health in maternity care: methods for meaningful stakeholder engagement

**DOI:** 10.1080/17482631.2023.2205282

**Published:** 2023-04-26

**Authors:** Kelly A. Umstead, Carolina Gill, Marina S. Pearsall, Alison M. Stuebe, Kristin P. Tully

**Affiliations:** a Department of Graphic, Experience, and Industrial Design, North Carolina State University, Raleigh, NC, USA; b Gillings School of Global Public Health, The University of North Carolina at Chapel Hill, Chapel Hill, NC, USA; c Department of Obstetrics and Gynecology, School of Medicine, The University of North Carolina at Chapel Hill, Chapel Hill, NC, USA

**Keywords:** Human-centered design in healthcare, social determinants of health, participatory workshops, maternity, perinatal, equity

## Abstract

**Purpose:**

The screening process for social determinants of health (SDoH) includes questions regarding life circumstances and barriers to accessing health care. For patients, these questions may be intrusive, biased, and potentially risky. This article describes human-centered design methods to engage birthing parents and health care team members around SDoH screening and referral in maternity care.

**Methods:**

Three phases of qualitative research with birthing parents, health care teams, and hospital administrators were conducted in the United States. Shadowing, interviews, focus groups, and participatory workshops addressed the explicit and tacit concerns of the stakeholders regarding SDoH during maternity care.

**Results:**

Birthing parents wanted to be informed of the purpose of the clinic collecting SDoH information and how this information is used. Health care teams want to feel they are providing reliable and quality resources to their patients. They would like greater transparency that administrators are acting on SDoH data and the information is reaching people that can assist patients.

**Conclusion:**

As clinics implement patient-centered strategies for addressing SDoH in maternity care, it is important to include patients’ perspectives. This human-centered design approach advances understanding of knowledge and emotional needs around SDoH and offers insights to meaningful engagement around sensitive health data.

## Introduction

A human-centered design (HCD) approach is characterized by promoting meaningful participation of stakeholders in both framing problems and developing solutions. Human-centerd design is empathy-driven and places emphasis on inductively synthesizing information and ideas from diverse sources in search of stakeholder-considered solutions. Human centerd-design researchers may use observations, interviews, and focus groups to develop frameworks, concepts, and prototypes of interventions that can be tested with end-users for improvement and refinement (Erwin & Krishnan, 2016). These activities can be conducted with stakeholders early in the innovation process to determine the root cause of priority issues and also in later stages of collaboration to generate solutions to problems (Roberts et al., 2016).

In contrast to the paradigm of research participants as “subjects,” HCD is consistent with stakeholder-driven research. Questions and activities are structured to solicit participant perspectives as partners in the discovery process, not to evaluate previously developed ideas. Centering stakeholder insights is promising in fields in which context, motivations, and experiences contribute to improved outcomes, such as in healthcare systems (Melles et al., 2021; Morrison & Dearden, 2013). Subsequently, HCD is increasingly utilized by health care quality improvement team members, researchers, and organizations as an effective strategy for addressing complex healthcare issues (Melles et al., 2021). HCD brings an empathetic approach to understanding people’s journeys through care systems where the focus on user-driven insights is effective for identifying multi-level interventions. In addition to medical device development, HCD has been successfully applied to healthcare management (Roberts et al., 2016) and the education of health care workers, public health practitioners, and medical professionals (Sandhu et al., 2015; van de Grift & Kroeze, 2016). Major healthcare organizations in the United States, including Kaiser Permanente and Mayo Clinic have begun establishing their own internal innovation units that incorporate design thinking and human-centered practices into their clinical settings (Holeman & Kane, 2020; Persson, 2017; Vechakul et al., 2015; Zuber & Moody, 2018).

### Social determinants of health in maternity care

Circumstances such as where people live, work, socialize, and learn, as well as the power structures that underlie them are related to certain health outcomes. These social or structural determinants of health (SDoH) include access to and receipt of quality health care, with intersecting structural, political, and social forces contributing to health outcomes (Crear-Perry et al., 2021; Sharpe et al., 2010). Within maternity health care, SDoH screening and referral is a tool for supporting the health of both birthing parents and their infants. Pregnancy and postpartum is a critical period for the short and long-term health of birthing parents (Bartick et al., 2017; Louis & Saade, 2015). Further, maternity patients’ experiences in systems of care influence their future health care engagement and utilization (Attanasio et al., 2021). For the fetus/infant, health outcomes are influenced by genetics, family social circumstances and emotions, environmental exposures, and caregiver behavioral patterns. Overall, the quality of health care services during this important part of the life course affects outcomes across generations.

To promote equity in maternity care services and outcomes, the American College of Obstetricians and Gynecologists have several recommendations. For example, health care team members should ask expectant patients about SDoH and record their strengths and vulnerabilities (American College of Obstetricians and Gynecologists, 2018). Health care providers should also actively refer patients to resources (American College of Obstetricians and Gynecologists, 2021). However, there are barriers to successful implementation of these best practices. First, screening of SDoH is not always universally conducted, and responses may not be clearly documented or easily accessible. Additionally, maternity clinics may have varying levels of SDoH resources available, and health care team members might not be aware or otherwise prepared to refer patients to these resources. Research findings suggest that a vast majority of health care practitioners are not confident in their ability to address the social needs of their patients (Robert Wood Johnson Foundation, 2011). Therefore, this study adopted a human-centered design approach to stakeholder engagement around social determinants of health in maternity care. The objective of this article is to outline research methods utilized in this project to advance understanding of SDoH screening and referral needs from stakeholder perspectives. The intentional, iterative approach can inform methods for partnering with stakeholders around sensitive health data, to advance knowledge on quality maternity care and other health care contexts.

## Method

**Figure 1.**
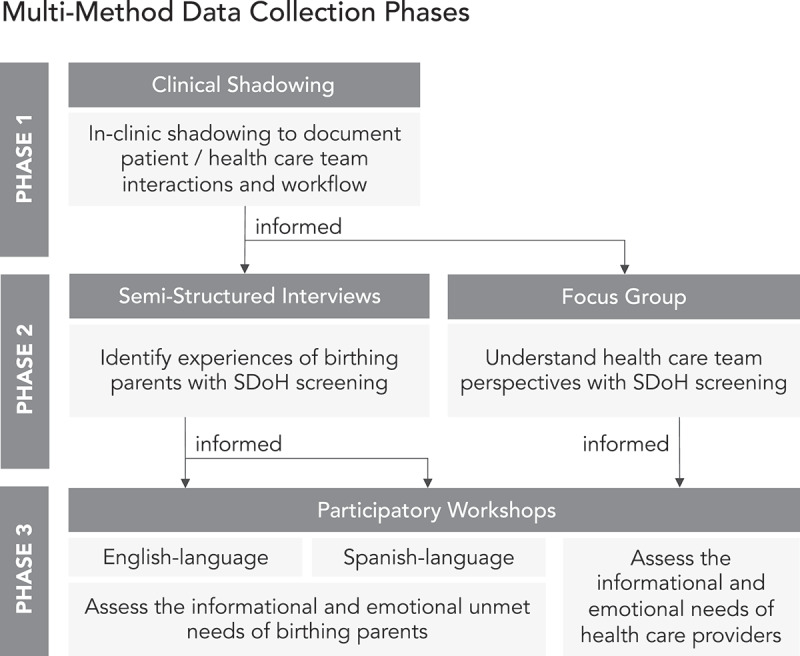
Multi-method, iterative data collection structure.

The study was reviewed by the University of North Carolina at Chapel Hill’s Institutional Review Board and determined to be exempt (IRB #18–2811). The goal of the research was to identify factors that could strengthen SDoH screening and referral from patient and health care team member perspectives, with an initial focus on optimizing clinical workflow. The multi-method data collection utilized a human-centered design approach developed in iterative phases. Three phases of qualitative research were conducted from December 2018 to January 2020, with the results from each phase informing the structure and content of the next data collection method (Figure 1). This approach was selected to build understanding and synthesize the complex needs around SDoH in the context of maternity care. Shadowing, interviews, focus groups, and participatory workshops collectively addressed the explicit, and more importantly, tacit priorities and concerns of the stakeholders. Participants included birthing parents and health care team members and administrators affiliated with a prenatal clinic of a university teaching hospital in the southeastern United States. Thematic analysis was conducted on field notes, transcriptions, and other artefacts, along with quantification of dot voting activities to identify priorities. Researchers used a collaborative selective coding process to cluster categories into themes, with consensus reached through comparison, iteration, and discussion. The themes identified in each research phase were evaluated in more depth in the subsequent round of data collection, as presented in Figure 1.

### Phase 1: Clinical shadowing

The multidisciplinary research team initiated the project by discussing objectives and establishing a shared understanding about their perspectives of strengths and unmet needs regarding SDoH screening, informed by the literature. The team strategized ways to obtain new knowledge, challenge assumptions, and compare baseline understandings to the maternity clinic observations. The aim of this research phase was for the non-clinical researchers to familiarize themselves with the maternity clinic environment, to assess workflows, identify existing resources, and observe SDoH-related information exchanged between patients and various health care team members.

#### Data collection

Two non-clinical researchers shadowed two obstetricians in the maternity clinic for four hours (eight total hours of observations). The shadowing included seven physician-patient interactions, presence with four nurses, one social worker and a conversation with a financial advisor. Researchers wrote observations onto study-developed documents that used an organizational framework of the following taxonomy: activities, environment, interactions, objects, and users (Martin & Hanington, 2012). The researchers also collected clinic materials (i.e., informational pamphlets) that were routinely provided to patients. The two researchers met for a debriefing session at the conclusion of their observation period, summarized observations and shared with the larger multidisciplinary research team.

#### Results

The clinical shadowing provided insight to the context of SDoH screening and referral, and categories of clinical practices and language were grouped into themes of: clinician preparation to see the individual, setting an agenda, building comfort and trust, motivational interviewing, non-judgemental communication, demonstrating empathy and respect, and harm reduction. Key phrases observed included “Let me tell you what I know about you, and you tell me what I’ve missed,” “We do this screen with moms because this is common,” and “We’re not here to force you to do anything—we want to work with what works for you even if that takes time.”

Findings revealed complex interactions between patients and the health care team members and within the health care team. The clinic offered multiple resources for birthing parents and families including in-clinic financial advising, access to social and government service representatives, hospital transportation services, and food pantry services. Access to these programs could be facilitated by health care team members. However, barriers to access included fragmented clinician awareness of these resources, including what supports are available, where to find details on the programs, and uncertainty around how to connect patients to these resources. Health information resources were shared with patients through multiple informational documents, but the material was dense and included an extensive list of resources that might or might not be relevant to the birthing parent. Much of the material was available in Spanish, but there were notable differences in the quality of the materials. For instance, the English brochures were printed in color and of professional print quality. The Spanish brochure was offered in greyscale and printed on a standard office printer. Furthermore, some of the Spanish translations were not accurate.

### Phase 2: Semi-structured interviews and focus group

To solicit stakeholder perspectives on the multiple aspects of the SDoH screening and referral as a part of maternity care, the research team conducted: 1) semi-structured interviews with birthing parents and 2) a focus group with health care team members.

### Semi-structured interviews with birthing parents

#### Data collection

The aim of interviews was to understand experiences of birthing parents regarding SDoH screening and referral in maternity care. The research team developed semi-structured interview guides in English and Spanish informed by the observations. Three general areas of inquiry were pursued: 1) SDoH screening and referral logistics­– to determine what SDoH questions had they been asked, by whom, and what resource information was provided; 2) SDoH information use and data privacy—to explore birthing parent views of why it might be (or might not be) helpful for them to share SDoH information; and 3) communication preferences—to determine birthing parents’ preferred communication strategies for aligning resources with their needs. All interviews were audio-recorded, professionally transcribed, and translated into English, where applicable.

As described in detail elsewhere (Tully et al., 2022), the research team conducted fifteen (15) semi-structured interviews with birthing parents. Nine of the birthing parents were English-speaking and six of the birthing parents were Spanish-speaking. All birthing parent participants were over the age of 18, had experienced maternity care within the last 12 months, and lived in the southeastern region of the United States. Participants were recruited from a University of North Carolina at Chapel Hill’s listserv, social media posts, and a local community center. English-language interviews were conducted via telephone by one non-clinician research team member and Spanish-language interviews were conducted via telephone by a native-speaking non-clinician research assistant. Example questions included: Were you asked about your ability to make and attend your prenatal appointments? How might these types of questions around SDoH impact your experiences of pregnancy and birth? If you needed more information or resources on these types of issues, how would you like to receive that?

#### Results

Through the interviews, birthing parents shared joys and stressors they faced during pregnancy, birth and postpartum. Common concerns were the limited knowledge of resources available to support their specific circumstances in advance of need, haphazard discovery of service options, and lack of clarity regarding clinical use of their SDoH responses (Tully et al., 2022). Further, two high-level themes inductively emerged: informational needs and emotional needs of the birthing parents regarding SDoH screening and referral in maternity care (Table I). The informational needs of the birthing parents reflected their desire to better understand why clinics are collecting SDoH information about them, how the information might be used in the healthcare setting and beyond, and benefits and/or consequences of sharing this sensitive information. The emotional needs of the birthing parents reflected the birthing parents desire to be seen, heard, and respected during their health care encounters.

**Table I.** Informational and emotional needs of birthing parents collected from interview responses.

Birthing Parent: Informational and emotional needs to address SDoH in maternity care.	
*I need to know*…	*I need to feel*…
if any of my SDoH answers can be used against me or my family.*	confident about my medical care.*
why the clinic is collecting SDoH information.*	that the information I am providing is acknowledged.*
how my SDoH answers are going to be used to help me and my family.*	respected.*
how the clinic is going to follow-up with me on my SDoH answers.*	comfortable communicating my concerns.*
if my SDoH information impacts my care.	included in health care decision making.* (written-in by participants)
how long my SDoH information will be stored in my medical record.	my concerns are taken seriously and acted upon.* (written-in by participants)
who sees my SDoH information.	that the clinic understands my reality.
if my responses in the medical record are accurate.	that I am being trusted.

* Needs prioritized by participants in the phase 3 workshop rating activity.

### Focus group with health care team members

The purpose of the focus group with maternity health care team members was to understand their perspectives regarding SDoH screening, resource awareness, patient care responsibilities in relation to SDoH, and the workload context.

#### Data collection

The health care team member focus group guide included addressing birthing parent priorities identified through the interviews. Eleven individuals participated and they held the following roles: clinic manager, pregnancy care manager, social worker, midwife, maternal fetal medicine physician, and nurse. The focus group activities were conducted in a private room in the clinic, facilitated by a non-clinician research team member and audio recorded for verbatim notetaking by the research team.

#### Results

The health care team members described wanting to have more clarity on SDoH resources so that they are able to provide relevant, quality, local resources to their patients. The clinicians expressed concern in being equipped to respond to their patients SDoH needs in meaningful ways.

The focus group data were analysed to identify unmet needs of the health care team members and structured around informational needs and emotional needs (Table II). The informational needs of the health care team were to know how the clinic is using SDoH information, to know that the information can be used in a way that is beneficial to patients, and best practices surrounding the collection of SDoH information. The emotional needs of the health care team were to feel comfort and confidence that the SDoH information they are sharing will positively impact their patients and to be better equipped to provide resources and act on patient responses.

**Table II.** Informational and emotional needs of health care team members collected from the focus group.

Health Care Team: Informational and emotional needs to address SDoH in maternity care.	
*I need to know*…	*I need to feel*…
that our clinic has specific resources to address SDoH concerns on site.*	that the clinic provides good quality SDoH resources.*
that SDoH information is getting to people that can respond to it.*	that I am being trusted by my patients.*
that the clinic is acting on SDoH information.*	comfortable communicating accurate SDoH related information.*
how to match patient needs to the appropriate resources.*	confident knowing the range of SDoH resources my clinic provides.*
that I can communicate information through the EMR system in an unbiased way.*	empowered to help patients around their SDoH challenges.*
how the clinic is going to follow-up with patients around SDoH issues.*	that I am being supported by the clinic (healthcare team).*
how often to revisit the SDoH questions.*	comfortable introducing SDoH screening.
why the clinic is collecting SDoH information.	
that SDoH information is being collected at a time that the clinic can be proactive.	
if my interpretation of the patient’s priorities or concerns is accurate.	
how SDoH answers are going to be used to help the patients.	
that I am trained to manage SDoH responses appropriately.	

* Needs prioritized by participants in the phase 3 workshop rating activity.

The concerns and sentiments from the focus group participants became the basis for the health care team workshop, which was designed for participants to have open conversations around the birthing parents’ accounts of informational and emotional needs and to prioritize the areas of focus.

### Phase 3: Participatory workshops

The purpose of this phase of the research was to co-create a patient-centered and clinically feasible path forward to address informational, emotional, and logistical needs for more effective SDoH screening and referral in maternity care. Participatory workshops were selected to provide an environment where shared experiences could be acknowledged, and to build trust and compassion around participants generating solutions together.

#### Data collection

Phase 3 research included the design and implementation of three participatory workshops: two birthing parent workshops and one health care team workshop. The birthing parent workshops consisted of 1) a group of eleven English-speaking birthing parents (four of which were also health care team members) convened at a children’s museum, and 2) a group of four Spanish-speaking birthing parents convened at a community centre. The workshop for health care team members consisted of 10 participants and was held in the clinic.

### Participatory workshops with birthing parents (in English and Spanish)

Birthing parent English-language workshops included an introduction, a maternity experience mapping activity, and card sorting activities. The Spanish-language workshop was structured in the same manner and led by a native Spanish-speaking non-clinical investigator.

#### Maternity care experience mapping

Experience maps are artefacts created by participants utilizing materials, images, and words designed by researchers. The process of making artefacts, such as these maps, enables people to access and express their past experiences and stimulates the articulation of desired experiences (Stappers & Sanders, 2003). The purpose of the experience map activity in this study was to promote participant reflection of their experiences throughout maternity care. Engaging in the activity was intended to encourage participation, sharing, and to build trust amongst workshop participants and facilitators.

**Figure 2.**
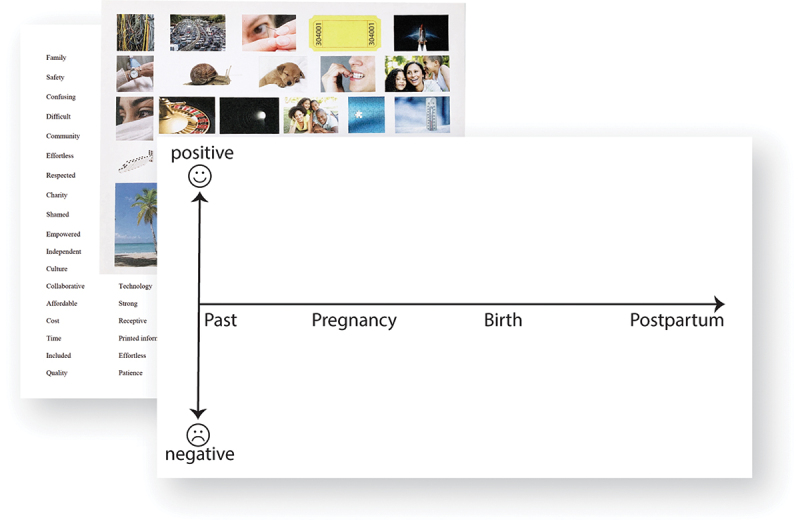
Maternity care experience mapping activity with stickers of images and words to be placed on the timeline.

Each participant was provided a timeline template which spanned pre-pregnancy through postpartum (Figure 2). They were asked to reflect on their thoughts, feelings, and experiences and place stickers of images and words along the timeline to represent these perspectives. Stickers included images intended to evoke responses like time, confusion, or shock by using images like a watch, a puzzle piece, and a lightning bolt, as well as providing descriptive words (such as “community,” “shamed,” or “empowered”) to help participants share their experiences. Participants were asked to write short descriptions or stories about their selected sticker images and words on the timeline. This experience mapping activity focused on emotional experiences, with participants encouraged to freely share their accounts. The generated maps were individually shared by each participant within their small groups (3–4 individuals) and, in the case of the English language workshop, summarized with the larger group.

#### Card sorting activity

**Figure 3.**
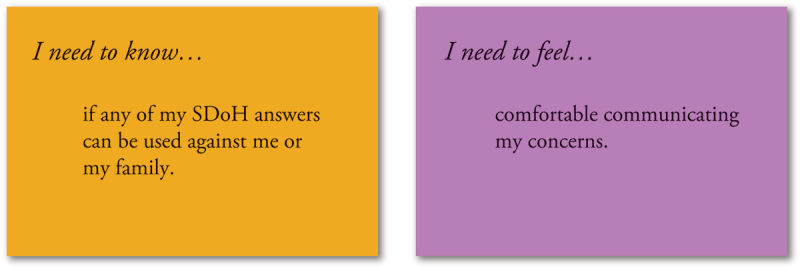
Example cards from the birthing parent sorting activity which included informational and emotional need statements.

The second activity for birthing parents was a card sorting exercise designed to enable the participants to articulate and prioritize desired experiences. Data points and themes identified from observations, interviews, and focus groups were written on colored index cards, and categorized as informational or emotional needs (Figure 3).

The informational based needs began with “I need to know … ” Example informational statements for birthing parents included “I need to know why the clinic is collecting SDoH information” and “I need to know who sees my SDoH information.” Emotional needs began with “I need to feel … ” For example, these statements included “I need to feel respected” and “I need to feel confident about my medical care.” Write-in responses were also encouraged through the provision of additional, blank cards. Birthing parents were provided with six emotional needs cards and asked to prioritize their top three needs. This activity was completed in small groups of 3–4 participants in the English language workshop and one group of 4 in the Spanish language workshop. The English and Spanish language workshops used the same informational and emotional needs statements written in the appropriate language. Upon selection of their top needs, participants were asked to discuss how those needs might be addressed. This process of prioritization and discussion was repeated using nine informational needs cards. The priority cards were placed on posters by the investigators for full group review. Participants then voted for their priorities by placing dot stickers next to cards.

#### Participatory workshop with health care team members

The health care team member workshop was conducted on site in a conference room in the obstetric clinic. The purpose of this workshop was to share findings from the health care team focus group, prioritize the needs of health care professionals around SDoH screening and referral, and elicit information on roles and responsibilities. This workshop included an introduction, a participatory card sorting exercise, and an activity to match roles with specific SDoH care responsibilities.

#### Card sorting activity

The card sorting activity was conducted in the same manner as the birthing parent workshop, where the emotional and informational needs cards were customized to health team member experiences. Example emotional need statements for the professionals included, “I need to feel comfortable introducing SDoH screening” and “I need to feel that the clinic provides good quality SDoH resources.” Example informational needs statements included, “I need to know how to match patient needs to the appropriate resources” and “I need to know how SDoH answers are going to be used to help patients.” Again, write-in responses were also encouraged through the provision of additional, blank cards. The health care team members were placed in small groups of approximately three individuals, and each group was provided seven emotional needs cards and twelve informational needs cards for review and prioritization. The groups were asked to reflect on the content, select their top three needs and discuss how those needs may be supported. Each group placed their top needs statements on a poster, along with their recommendations for implementation. To conclude the activity, the small groups shared their findings with the large group and each participant was given three dot stickers to identify points they considered to be priorities.

#### Roles and responsibilities activity

In the clinical workshop with health care team members, the second activity instructed the health professionals to rank the roles of those who should be responsible for SDoH screening, connecting patients with resources, referral follow up, SDoH resource coordination, and workflow integration. Each participant was asked to individually assign points to the roles they believed were most responsible for the tasks (Table III). The documents were then collected by the investigators for analysis.

**Table III.** Tasks and roles regarding SDoH care responsibilities in the clinic.

Tasks:		
Inquiring about SDoH with the patients is the responsibility of:		
Connecting patients with resources is the responsibility of:		
Communicating strengths of patients is the responsibility of:		
Following up with patients regarding referrals is the responsibility of:		
Acquiring SDoH resources for the clinic is the responsibility of:		
Incorporating patient’s SoDH challenges into clinic workflow is the responsibility of:		
Roles:		
Pregnancy or care manager	Nurse	Midwife
Obstetrician	Office Personnel	Social Services
Financial Office Personnel	Patients	Other (specify)

#### Results

**Figure 4.**
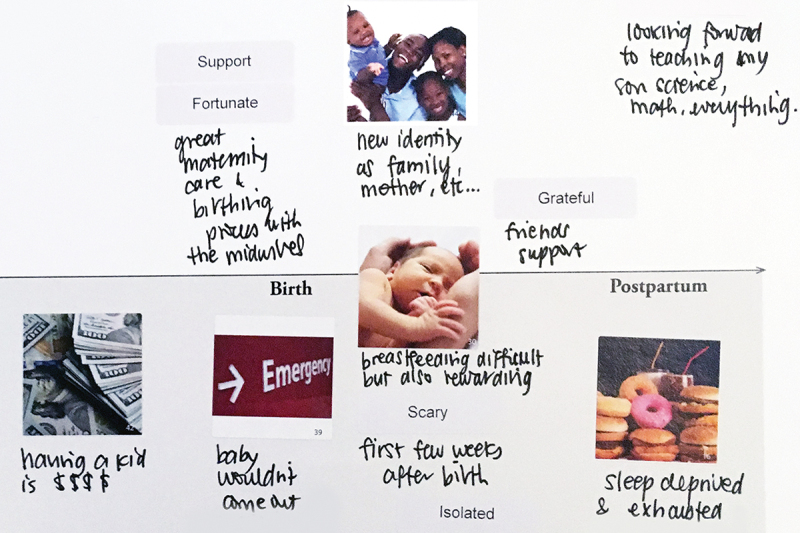
Sample of experience map depicting imagery, words, and handwritten notes.

Birthing parents created maps to depict their personal experiences around childbirth (See Figure 4). Using the maps as prompts, they shared feelings of isolation, loss, discrimination, and neglect alongside positive feelings of joy with their baby, good relationships with midwives, and community support. These negative and positive experiences occurred along their maternity journeys, often with negative and positive emotions at the same time points. When sharing their experience maps, Spanish-speaking birthing parents discussed instances when medical procedures were scheduled and or performed without the birthing parent having a clear understanding of the procedure or the associated medical necessity. They also reported times where they did not have an interpreter present and were unable to ask questions of their health care providers or pursue clarification due to lack of accommodation for limited English-language proficiency.

The card sorting exercise from the English language workshop prioritized birthing parent needs, as identified with asterisks in Table I. Birthing parents wanted to know how and why SDoH information would be used and requested appropriate integration, documentation, and policies surrounding the collection of this information. Participants expressed a need to feel respected and did not want SDoH collection to trigger discrimination. Also, participants expressed a need to be included in decision making and wanted to have trust and confidence in their providers. Moving forward, birthing parents suggested that bias training is essential for health care team members, so patients could be better supported in dealing with their challenges.

The Spanish language workshop data reflected these same themes (Table IV) and additionally foregrounded inequities in care. During discussion of the card sorting exercise, the participants expressed the need for language concordant care and compassion.

**Table IV.** Prioritization of needs during Spanish speaking birthing parent workshop.

Spanish Speaking Birthing Parent: Informational and emotional needs to address SDoH in maternity care.	
*I need to know…*	*I need to feel…*
why is the clinic collecting information regarding SDoH.*	confident about my medical care.*
who has access to the information related to my SDoH.*	comfortable communicating SDoH related information.*
how the clinic is going to follow-up with me on my SDoH answers.*	that the clinic understands my reality.*
how my SDoH answers are going to be used to help me and my family.*	that the information I am providing is acknowledged.*
if any of my SDoH answers can be used against me or my family.*	that I am being trusted.
how long my SDOH information will stay in my record.	
if my SDoH will have an impact in the care I receive.	

* Needs prioritized by participants in phase 3 workshop rating activity.

The needs of the health care team members were different from those of the birthing parents. The card sorting exercise results emphasized the importance of shared accountability, integration of SDoH screening into the workflow, and the need for administrative support to properly respond to patients’ needs (Table II). As detailed elsewhere (Tully et al., 2022), key suggestions from the participants to address these unmet informational needs included: having more in-person (outpatient) support within clinics and having a liaison for resources.

In the roles and responsibilities activity, the clinical care team identified that obstetricians, nurses, and pregnancy care managers should play a central role in SDoH screening and referral in maternity care. Most of the health care team members reported that pregnancy care managers should be responsible for activities including connecting patients to resources, following up with the patients regarding referrals, and affirming patient strengths. Several respondents emphasized the importance of clinical leadership in coordinating SDoH resources for the site, as well as managers being a resource for workflow integration.

## Discussion

This study engaged birthing parents and health care team members to document their experiences regarding SDoH in maternity care. The methods presented in this article describe a human-centered design approach, which utilized multiple data collection methods over three phases of research to comprehensively address the SDoH logistics, workflow, and stakeholder needs (see Box I). Synthesized data from each research phase informed the subsequent approach, culminating in participatory workshops with English- and Spanish-speaking birthing parents and health care team members. The workshop participants prioritized informational and emotional needs around SDoH clinical screening and referral processes.

**Table:** **Box I.** Methods for meaningful stakeholder engagement in the context of sensitive health data.

*Phase 1*. Observations, such as clinical shadowing, are important to determine the context of health interactions. Workflows, existing resources, and information exchange between patients and health care team members were identified as core components of care for clinical screening and referral for social determinants of health. *Phase 2*. To determine perspectives of stakeholders engaged in SDoH care, interviews and focus groups were utilized to determine aspects of engagement that matter most to birthing parents and health care team members. *Phase 3*. Once stakeholder priorities were known, the researchers and participants partnered through workshops to engage in activities designed to elicit reflections on the complexities uncovered through data collection and develop strategies for system improvements.

Birthing parents wanted to be informed of the purpose of maternity clinics collecting SDoH information, to be told how this information would be used, and to know that sharing responses would assist in getting their needs met. The birthing parents wanted to feel confident in their medical care, which included trusting their health care providers and feeling respected by them. Health care team members requested more on-site resources to provide to their patients, greater transparency that their administrators are acting on collected SDoH data, and to know that the response information is reaching the people that can assist the patients. Health care team members would like to feel they are providing up-to-date, reliable, and quality resources to their patients. See Box II.

**Table:** **Box II.** Human-centered design workshop findings on birthing parent and health care team member priorities to regarding screening and referral for SDOH in maternity care.

Birthing parents described needing to know how their SDoH screening responses might be used, both against them or to help them. They need to feel confident about their care, acknowledged, and respected. Along the pre-pregnancy through postpartum continuum, birthing parents had negative and positive experiences, including at the same time points. Desired experiences include compassion, language concordance, and non-discrimination. Health care team members need to know SDoH resources to address, that patient information is getting to the appropriate person for response. They need to feel aware of quality resources, trusted by patients, and comfortable communicating accurately around SDoH. For SDoH in maternity care, health care team members wanted more support, coordination, and awareness of resources, with clinical leadership and managers coordinating SDoH resources.

This study was designed to be iterative, and the research process remained flexible throughout the project to allow for reflection, validation, and adaptation to new knowledge. Each phase of engaging with stakeholders increased the understanding of SDoH screening and referral in maternity care. The human-centered approach was evident in multiple facets of the work, from the study environment to the designed activities. Careful consideration of the context was critical for the participants to have meaningful engagement in activities. These strategies included appropriate venues that were accessible and encouraged participation of mothers with their nursing infants. To design useful and targeted workshops, the researchers invested time in the early research phases to understand the complexity of the environment through observations, interviews, and focus groups. These findings helped to focus the workshops to an appropriate scope, knowing that the larger context of SDoH is complex and filled with interdependencies within the healthcare ecosystem.

The experience mapping activity served as an important complement to the interviews and observations, especially with the Spanish-speaking participants. This activity encouraged these participants to share more information in a communal setting and all the participants were engaged in the experience mapping.

As clinics strive to implement patient-centered strategies for addressing SDoH in maternity care, it is important to include and collaborate with birthing individuals. The human-centered design approach centers stakeholders’ voices, values their experiences, and is structured to identify their expressed and tacit needs. The research activities in this study were designed to engage birthing parents and health care team members as active knowledge generators, and to appreciate their respective expertise. Through this empathic approach, the researchers gained novel insight of how to effectively address SDoH in clinical care as reported here and with complementary publication (Tully et al., 2022). In maternity care and beyond, human centered-design can incorporate user-driven insights to question the status quo, bring light to systems that sustain health inequities, and offer novel opportunities to improve health and wellbeing across generations.
